# A novel variant in *SIAH1* associated with autosomal dominant Buratti-Harel syndrome

**DOI:** 10.3389/fnins.2025.1677646

**Published:** 2026-01-07

**Authors:** Hong Zheng, Lan Zhang, Fuwei Li

**Affiliations:** 1 The First Affiliated Hospital of Henan University of Chinese Medicine, Zhengzhou, China; 2 School of Pediatrics, Henan University of Chinese Medicine, Zhengzhou, China; 3 Department of MRI, The First Affiliated Hospital of Henan University of Chinese Medicine, Zhengzhou, China; 4 Beijing Chigene Translational Medical Research Center Co., Ltd., Beijing, China

**Keywords:** Buratti-Harel syndrome, *de novo* variant, neurodevelopmental disorder, *SIAH1*, zinc-finger domain

## Abstract

**Introduction:**

Buratti-Harel syndrome (BURHAS) is a rare autosomal dominant neurodevelopmental disorder caused by *SIAH1* (Siah1 E3 ubiquitin ligase) variants, characterized by infantile hypotonia, global developmental delay, and variable multisystem involvement. To date, 13 pathogenic *SIAH1* variants have been reported in 13 patients, but the functional and phenotypic implications of mutations in the *SIAH1* zinc finger domain remain poorly characterized. This study included a Chinese pediatric patient with unexplained neurodevelopmental and multisystem abnormalities.

**Methods:**

Trio whole-exome sequencing (WES) was performed to identify potential causative variants. Structural modeling was used to analyze the impact of the identified variant on *SIAH1* protein structure. Additionally, a comparative genotype-phenotype analysis was conducted on 14 genetically confirmed BURHAS cases (including the present patient).

**Results:**

A *de novo* missense variant [c.288C > G (p.Phe96Leu)] in the zinc finger domain of *SIAH1* was identified in the patient. Structural modeling revealed that this variant destabilized the zinc finger domain (DDG = –2.09 kcal/mol), which may disrupt the ZnF-1 domain function by impairing zinc ion-mediated structural stability. Comparative analysis of 14 genetically confirmed cases (including this study) demonstrated a genotype-phenotype correlation: zinc finger domain variants (*n* = 4) exhibited broader phenotypic heterogeneity compared to RING domain variants (*n* = 7), which were enriched for severe developmental delay and reproductive anomalies.

**Discussion:**

This study expands the mutational spectrum of *SIAH1*-associated disorders. We advocate for the inclusion of *SIAH1* in diagnostic panels for patients with unexplained neurodevelopmental disorders and multisystem dysmorphisms.

## 1 Introduction

Siah1 E3 ubiquitin ligase encodes a RING-type E3 ubiquitin ligase that comprises an N-terminal catalytic RING domain, two zinc finger domains, and a C-terminal substrate-binding domain (SBD) (House et al., 2006). This enzyme critically regulates the Wnt/β-catenin signaling pathway by targeting Axin for ubiquitin-mediated degradation, thereby promoting β-catenin accumulation and transcriptional activation (Ji et al., 2017). Beyond its role in Wnt signaling, SIAH1 is essential for neuronal development through ubiquitination-dependent modulation of Akt3 turnover and synaptic proteostasis (Ko et al., 2019), and participates in metabolic and immune processes.

Pathogenic monoallelic variants in *SIAH1* underlie Buratti-Harel syndrome (BURHAS; MIM #619314), a neurodevelopmental disorder that remains rarely documented. To date, only two studies have established disease associations: Buratti et al. (2021) first identified heterozygous *de novo* missense variants in five individuals with core features of developmental delay, infantile hypotonia, and dysmorphism, demonstrating attenuated Wnt pathway activity due to loss-of-function mechanisms (Buratti et al., 2021). Subsequently, Douiev et al. (2025) expanded the phenotypic spectrum by reporting eight additional cases harboring six truncating and two missense variants, highlighting multisystem involvement including cardiac malformations, skeletal anomalies, and recurrent infections (Douiev et al., 2025).

Herein, we describe a sporadic case of BURHAS presenting with developmental delay, dysmorphic features, limb muscle weakness, and distinctive neuroimaging abnormalities, associated with a novel *de novo SIAH1* variant. This finding broadens the mutational spectrum of SIAH1 and further delineates the clinical heterogeneity of this syndrome.

## 2 Materials and methods

### 2.1 Subject recruitment

The clinical and genetic information of the patient was collected at the Department of Pediatrics in the First Affiliated Hospital of Henan University of Chinese Medicine. Written informed consent for genetic testing and data sharing was obtained from the patient’s legal guardians. The study protocol was approved by the Ethics Committee of the First Affiliated Hospital of Henan University of Chinese Medicine(approval number: 2025HL-669).

### 2.2 Whole-exome sequencing

Genomic DNA was extracted from peripheral blood leukocytes using the QIAamp DNA Blood Midi Kit (Qiagen, Hilden, Germany) following the manufacturer’s protocol. DNA integrity was confirmed by 1% agarose gel electrophoresis (A260/A280 ratio: 1.8–2.0), and quantification was performed using a Qubit 4 Fluorometer (Thermo Fisher Scientific, Waltham, MA, USA). Library construction was performed using the IDT xGen Exome Research Panel v2.0 (Integrated DNA Technologies, Coralville, IA, USA), targeting ∼39 Mb of coding regions and splice junctions. Hybrid capture was conducted according to the manufacturer’s protocol, followed by paired-end sequencing (PE150) on the BGI DNBSEQ-T7 platform (BGI-Shenzhen, China). Raw sequencing data were generated with an average depth of >120×, ensuring >98% target coverage at ≥20×.

### 2.3 Bioinformatics analysis

Raw sequencing reads underwent quality control using fastp v0.23.2 (Chen et al., 2018) to remove adapters and low-quality reads (Phred score < 20). Clean reads were aligned to the GRCh37/hg19 reference genome using Burrows-Wheeler Alignment-Maximal Exact Matches (BWA-MEM) v0.7.17 (Li and Durbin, 2009). Duplicate reads were marked with Picard v2.27.1 (Broad Institute), and variant calling was performed using Genome Analysis Toolkit (GATK) HaplotypeCaller v4.4.0 (Van der Auwera et al., 2013). Variants were annotated using the Chigene Cloud Platform (v2.3), integrating data from ClinVar (release 2023-05), gnomAD v3.1.2, and dbSNP v155. Functional impact predictions were derived from a consensus of Sorting Intolerant From Tolerant (SIFT) (score < 0.05), PolyPhen-2 (score > 0.85), Combined Annotation Dependent Depletion(CADD) PHRED (score > 20), and Rare Exome Variant Ensemble Learner(REVEL) (score > 0.75). Splice-site variants were evaluated using MaxEntScan, dbscSNV, and SpliceAI (threshold Δscore > 0.2). Pathogenicity classification adhered to American College of Medical Genetics and Genomics/Association for Molecular Pathology(ACMG/AMP) guidelines (Richards et al., 2015) using InterVar v2.0.1. The WES filtering steps are summarized in Supplementary Table 1.

### 2.4 Structural analysis

AlphaFold 3.0 predicts the wild-type (WT) and mutant structures of the protein. (Jumper et al., 2021) and visualized with PyMOL v2.5.2. Conservation analysis was conducted with Molecular Evolutionary Genetics Analysis X(MEGA X) (Kumar et al., 2018). We utilized the I-Mutant 2.0 program to predict the impact of the missense variant on protein stability (Capriotti et al., 2005). Alterations in protein stability were evaluated using the free energy change (DDG, kcal/mol), where a negative DDG value indicates a reduction in the stability of the mutant protein.

## 3 Results

### 3.1 Clinical presentation

**FIGURE 1:**
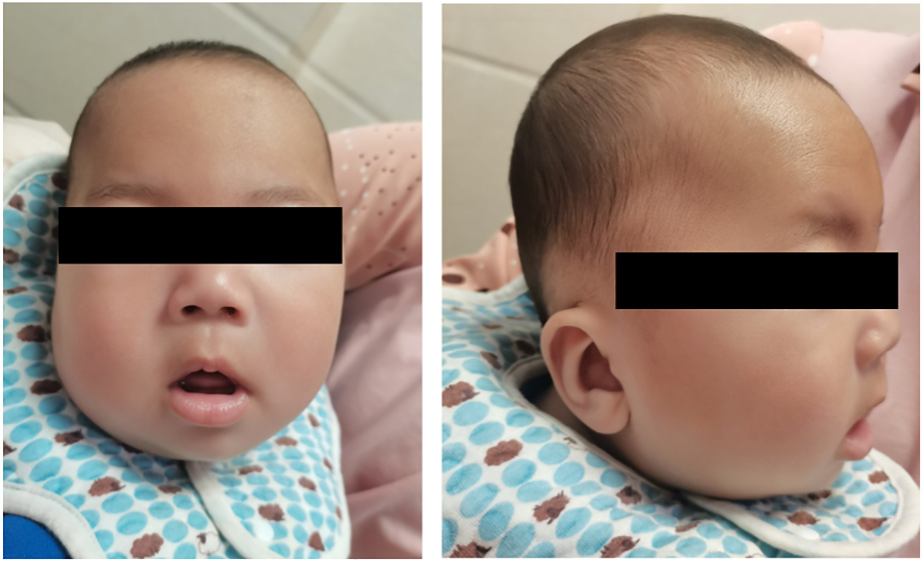
Facial photographs (frontal and lateral views) of the patient, demonstrating craniofacial dysmorphism, including Increased interpupillary distance, ptosis, blepharophimosis, low-set ears, microtia, bilateral asymmetry, broad nasal bridge, hypoplastic philtrum, abnormal mouth corner, high-arched palate.

**FIGURE 2:**
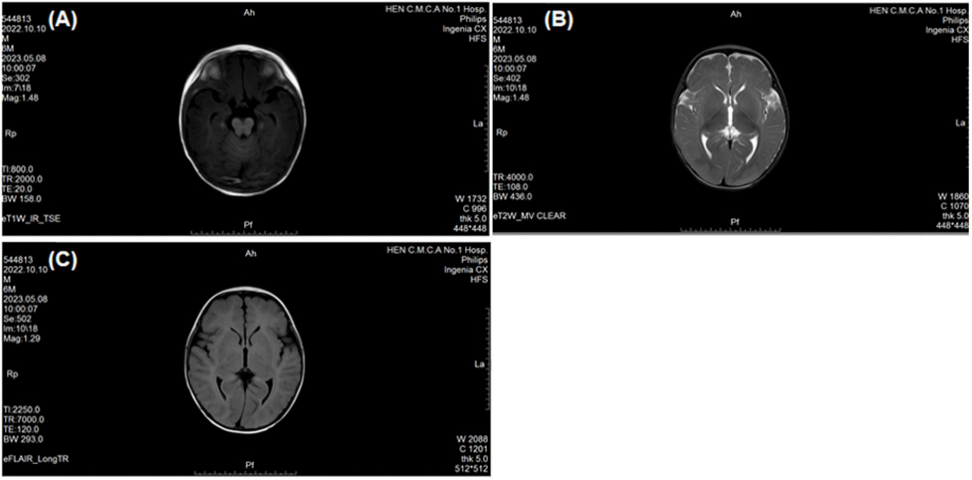
Brain MRI (3.0T) reveal: **(A)** Linear hypointense signal in the right frontal lobe (likely vascular shadow); **(B)** Bilateral frontotemporal subarachnoid space widening; **(C)** Bilateral otomastoiditis.

A 5-month-24-day-old male infant, delivered at full term via cesarean section due to oligohydramnios, exhibited a birth weight of 3.6 kg. The mother (age 40) and father (age 33) were healthy with no reported consanguinity. The patient had an older brother and sister, both developmentally normal. At 4 months of age, the infant presented with progressive neurodevelopmental delay characterized by: (1). Motor Developmental Delay: Impaired head control and truncal motor developmental delay; Hypotonia, adducted thumb deformity; Truncal hypotonia; Hyperreflexia. (2). Dysmorphic Features: Craniofacial dysmorphism: Increased interpupillary distance, ptosis, blepharophimosis, low-set ears, microtia, bilateral asymmetry, broad nasal bridge, hypoplastic philtrum, abnormal mouth corner, high-arched palate; Skin abnormality: Congenital dermal melanocytosis (Figure 1). (3). Neuroimaging Abnormalities: Brain Magnetic Resonance Imaging(MRI) revealed: Linear hypointense signal in the right frontal lobe (likely vascular shadow); Bilateral frontotemporal subarachnoid space widening; Bilateral otomastoiditis (Figure 2). (4). Outline laboratory data with abnormal findings are summarized in Table 1. Key abnormalities include hypoproteinemia, elevated alkaline phosphatase (ALP) and creatine kinase isoenzyme (CK-MB), reduced serum creatinine, hyponatremia, hyperphosphatemia, and decreased homocysteine. (5). Developmental Assessment: Bayley Scales of Infant Development III scores: Composite quotient: 43.5 (severe delay); Domain-specific scores: Adaptive behavior: 56.7; Gross motor: 33.4; Fine motor: 48.2; Language: 27.9; Social-emotional: 51.3. Alberta Infant Motor Scale grades: Prone: 3/5; Supine: 4/5; Sitting: 0/5; Standing: 0/5. During the most recent follow-up, the patient, who was 2 years and 8 months old, was receiving rehabilitation therapy at a local hospital. The psychological developmental quotient assessment report indicated the following scores: gross motor 75.0, fine motor 103.1, adaptive ability 84.4, language ability 40.6, and social behavior 70.3, resulting in a total developmental quotient of 74.7 and a mental age of 23.4 months. The evaluation results indicated slight developmental delay.

**TABLE 1:** Laboratory abnormalities and reference ranges.

Specific indicator	Detection result	Reference range
Total protein	48 g/L	60–80 g/L
Alkaline phosphatase (ALP)	320 U/L	45–125 U/L
Creatine kinase isoenzyme (CK-MB)	45 U/L	0–24 U/L
Serum creatinine	22 μmol/L	27–62 μmol/L
Sodium (Na^+^)	130 mmol/L	135–145 mmol/L
Phosphorus (PO_4_^3–^)	2.1 mmol/L	0.8–1.5 mmol/L
Homocysteine	4.2 μmol/L	5.0–15.0 μmol/L

### 3.2 Genetic findings

**FIGURE 3:**
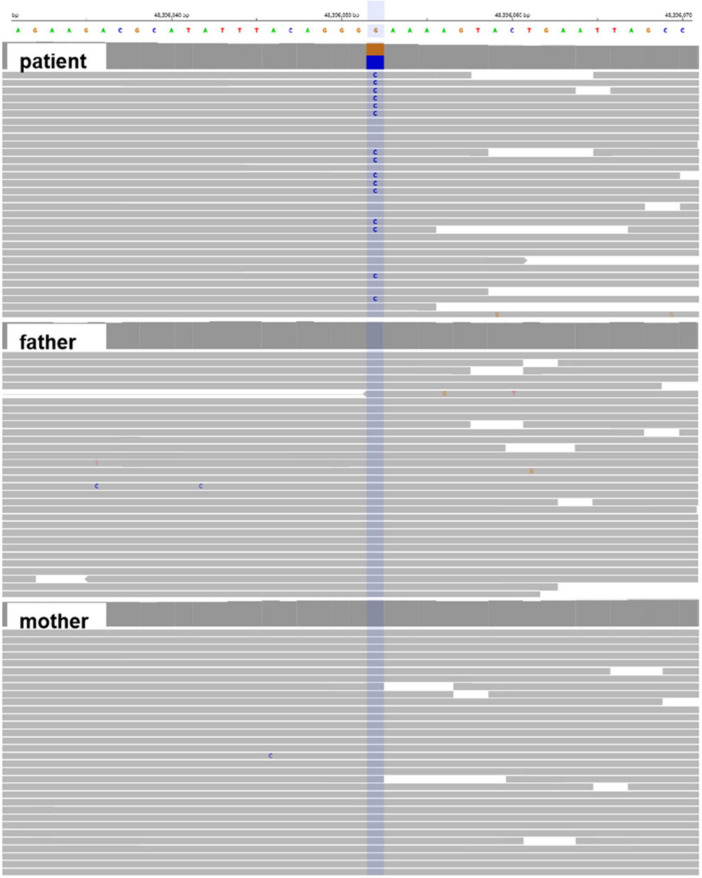
Whole-exome sequence data for the affected portion of the *SIAH1* c.288C > G visualized in integrative genomic viewer (IGV) demonstrating a heterozygous variant for the proband, whereas the wild-type sequence was observed in parents.

**FIGURE 4:**
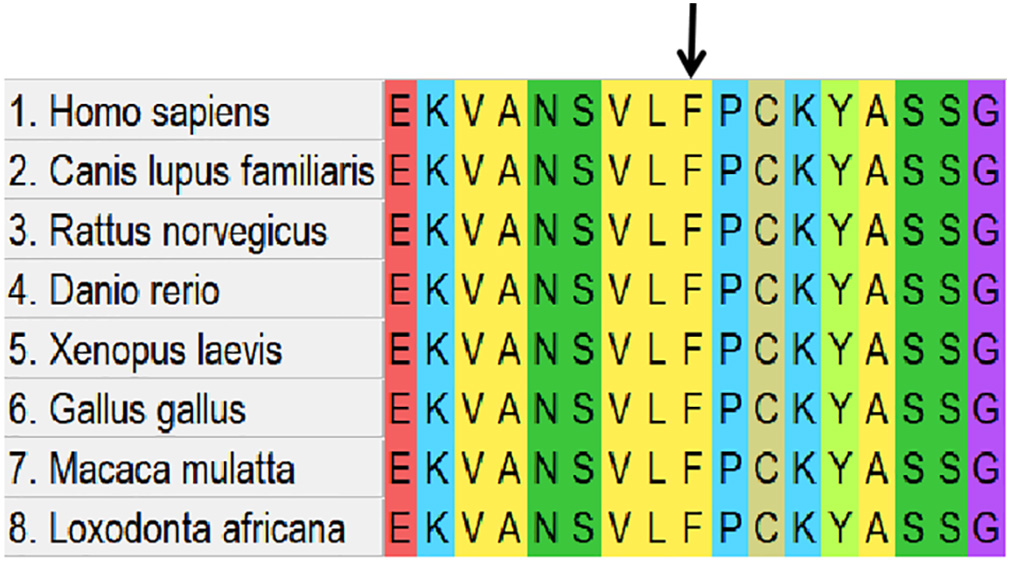
Conservation of the affected amino-acid residue across distinct species.

**FIGURE 5:**
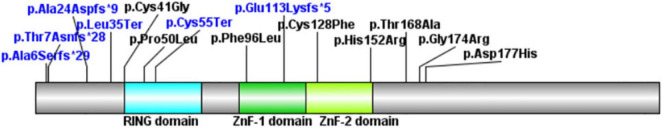
Schematic diagram of genetic variants and functional domains in SIAH1. This diagram depicts functional domains (blue RING domain, green ZnF-1 domain, yellow-green ZnF-2 domain). Truncating variants are labeled in blue font, and missense variants are annotated in black font.

**FIGURE 6:**
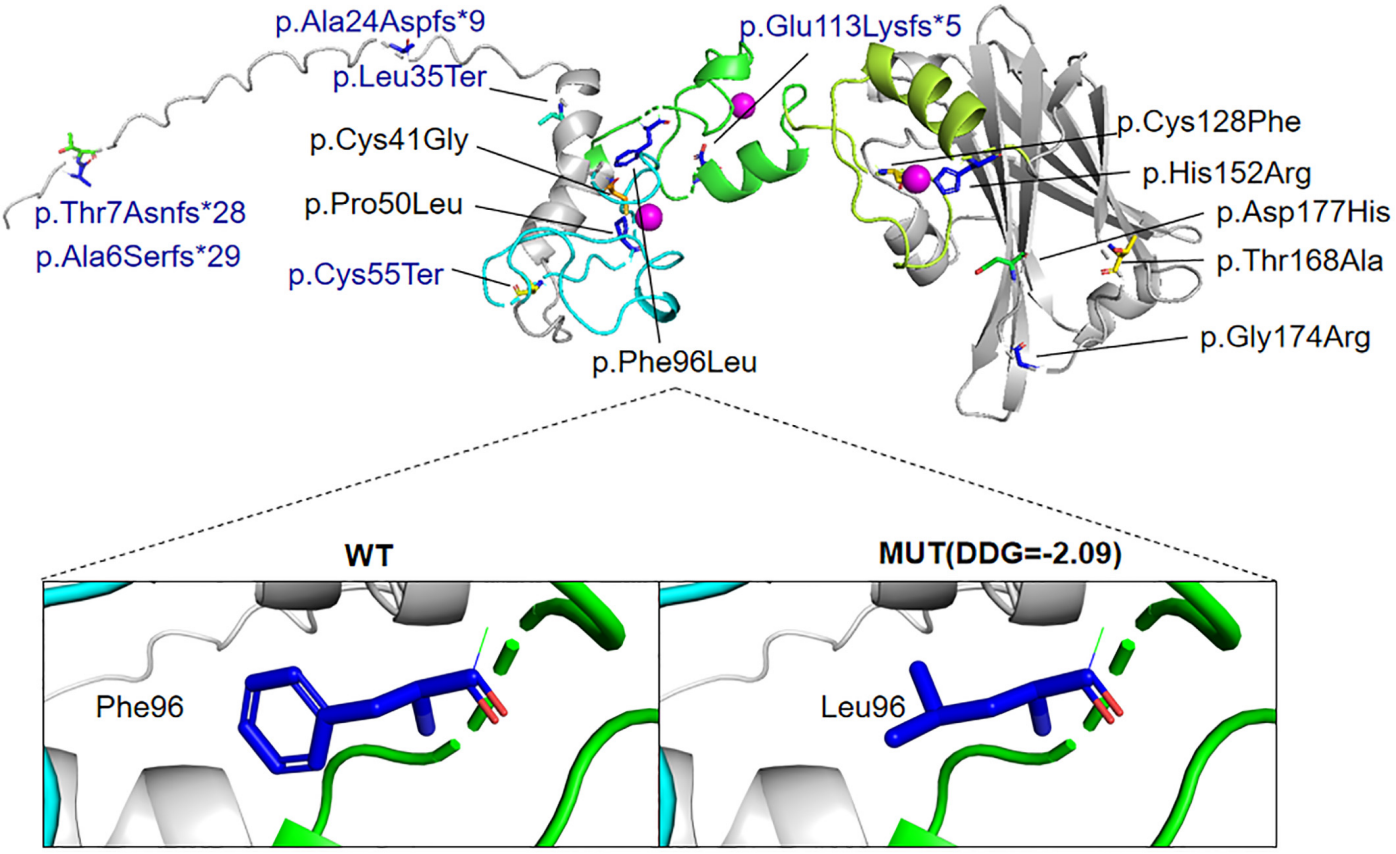
Three-dimensional structure: Location of all variants published variants in SIAH1 protein. Blue region, RING domain; Green region, ZnF-1 domain; Yellow-green region, ZnF-2 domain. Purple spheres, Zn^2+^. DDG, free energy change value, DDG < 0: decrease stability; MUT, mutant type; WT, wild-type. In the HGVS (Human Genome Variation Society) protein nomenclature guidelines, the symbol * represents a stop codon (the codon that signals the end of protein translation).

Whole-exome sequencing identified a *de novo* heterozygous missense variant in *SIAH1* (NM_003031.4): c.288C > G (p.Phe96Leu) (NC_000016.10:g.48362140:G > C). (1). Inheritance Pattern: Confirmed *de novo* origin by trio-WES (Figure 3); Wild-type alleles detected in both parents. (2). Molecular Consequence: Transition of cytosine to guanine at nucleotide 288; Non-synonymous substitution of phenylalanine (F) with leucine (L) at codon 96. (3). Novelty Assessment: Absent from population databases (gnomAD v3.1.2, 1000 Genomes, ESP6500, ExAC); Not reported in internal Chigene database. (4). Computational Predictions: Pathogenicity scores: SIFT: Deleterious (score 0.00); PolyPhen-2: Probably damaging (score 0.999); CADD PHRED: 28.7; REVEL: 0.89; AlphaMissense: Pathogenic (score 0.98). Conservation analysis: Phe96 is invariant across 12 vertebrate species (Figure 4). (5). Structural Impact: Located within the zinc finger domain (ZnF1) of SIAH1 (Figures 5, 6); The predicted template modeling (pTM) values of the wild-type (WT) and mutant structures are 0.69 and 0.68, respectively (range: 0–1; values > 0.5 indicate reliable protein folding). This variant is spatially proximal to the Zn^2+^ within the ZnF-1 domain, and may disrupt the function of ZnF-1 by impairing the zinc ion-mediated structural stability. (6). Destabilizes protein structure (DDG = −2.09 kcal/mol). Pathogenicity Classification: Classified as Likely Pathogenic (PS2, PM2, PP2, PP3) per ACMG/AMP guidelines.

## 4 Discussion

This study expands the phenotypic and mutational spectrum of *SIAH1*-associated neurodevelopmental disorders (NDDs) by reporting a novel *de novo* missense variant (c.288C > G, p.Phe96Leu) in a pediatric patient presenting with developmental delay, dysmorphic features, and brain imaging abnormalities. Combined with 13 previously reported cases (Table 2), our cohort analysis reveals a syndromic disorder characterized by systemic involvement, including intellectual disability (100%), speech/motor delay (100%), craniofacial dysmorphisms (100%), and multisystem anomalies (hypotonia 77%, ear abnormalities 79%, gastrointestinal involvement 78%, hand or foot abnormalities 77%, recurrent infections 45%, cardiovascular abnormalities 45%, genitourinary abnormalities 75%, and endocrine abnormalities 75%).

**TABLE 2:** Clinical findings in individuals with heterozygous Siah1 E3 ubiquitin ligase (*SIAH1*) variants.

Clinical feature	This study	Total [Buratti et al. (2021), Douiev et al. (2025)]
Gender	Male	7 female,7 male
Age at evaluation	5 months	5 months–21 years
Intellectual disability ID	Yes	10/10 (100%)
Speech delay	Yes	13/13(100%)
Motor developmental delay	Yes	14/14 (100%)
Abnormality of brain imaging	Linear hypointense signal in the right frontal lobe (likely vascular shadow); Bilateral frontotemporal subarachnoid space widening; Bilateral otomastoiditis.	3/9 (33%)
Hypotonia	Yes	10/13 (77%)
Laryngomalacia	No	9/14 (64%)
Abnormality of the digestive system	No	7/9 (78%)
Recurrent infections	NA	5/11 (45%)
Abnormality of the head	Craniofacial dysmorphism: Increased interpupillary distance, ptosis, blepharophimosis, low-set ears, microtia, bilateral asymmetry, broad nasal bridge, hypoplastic philtrum, abnormal mouth corner, high-arched palate	14/14 (100%)
Abnormality of the ear	Low-set ears, microtia, bilateral asymmetry	11/14 (79%)
Abnormal of hand and foot morphology	Adducted thumb deformity	10/13 (77%)
Abnormality of the Cardiovascular system	No	5/11 (45%)
Abnormality of the genitourinary system	No	6/8 (75%)
Abnormality of the endocrine system	No	3/4 (75%)
Others	Hypoproteinemia, elevated alkaline phosphatase, abnormality of creatine kinase isoenzyme, reduced serum creatinine, hyponatremia, hyperphosphatemia, decreased homocysteine	–

NA, not available.

### 4.1 Functional implications of *SIAH1* variants

The *SIAH1* gene, encoding a RING-type E3 ubiquitin ligase, plays a pivotal role in proteostasis through substrate ubiquitination. Buratti et al. (2021) reported five novel *de novo* missense variants in the *SIAH1* gene, and validated the pathogenicity and functional impact of *SIAH1* variants through Luciferase reporter gene assays and Axin degradation experiments. Luciferase reporter gene assay demonstrated that wild-type SIAH1 significantly activated the Wnt/β-catenin pathway, as evidenced by elevated STF signals, while the mutants (C41G, C128F, etc.) completely lost the ability to stimulate the pathway, indicating dysregulation of Wnt signaling. Furthermore, the Axin degradation assay showed that wild-type SIAH1 effectively reduced Axin protein levels, whereas the mutants were unable to degrade Axin. Additionally, some mutants (e.g., C41G, C128F) exhibited abnormal protein stability, with higher expression levels than wild-type, suggesting defects in their ubiquitination function. Douiev et al. (2025) reported six *SIAH1* truncating variants that are expected to result in the loss of key functional sequences, thereby predicting a partial or complete loss of protein activity. The probability of loss-of-function intolerance (pLI) score for *SIAH1* is 1, with an observed/expected (o/e) ratio of loss-of-function variants at 0.09 and a Z-score of 4.84 for missense variants. This indicates that SIAH1 is highly conserved within the general population and that there are nearly no tolerated loss-of-function variants, as supported by data from the gnomAD database. These findings support haploinsufficiency as the pathogenic mechanism underlying SIAH1 variants. The p.Phe96Leu variant is located within the zinc finger domain, may disrupt the function of ZnF-1 by impairing the zinc ion-mediated structural stability and alters SIAH1 protein function.

### 4.2 Comparative genotypic-phenotypic analysis

Compared to previously reported missense/non-sense variants, our case highlights the phenotypic variability associated with SIAH1 dysfunction. While truncating variants (non-sense/frameshift) tend to cluster in the RING domain and manifest with severe developmental delay (Buratti et al., 2021), missense variants in the zinc finger domain (including p.Phe96Leu) exhibit broader phenotypic heterogeneity, potentially reflecting domain-specific functional redundancy. Notably, our patient’s multisystem involvement (e.g., otomastoiditis, electrolyte imbalance) diverges from classical SIAH1-associated phenotypes, suggesting potential modifier genes or environmental interactions.

### 4.3 Study limitations and future directions

While this study advances our understanding of *SIAH1*-associated pathogenesis, several limitations warrant consideration: (1). Sample Size: The small cohort (*n* = 14) restricts genotype-phenotype correlation analysis. (2). Functional Validation: *In vitro* assays (e.g., Axin degradation, Wnt reporter assays) were not performed on the novel variant. (3). Mechanistic Insights: The role of *SIAH1* in non-canonical Wnt pathways or crosstalk with other signaling networks remains unexplored. Future studies should prioritize: Large-scale international collaborations to establish a centralized variant registry; Development of SIAH1 conditional knockout models to dissect tissue-specific roles; Integration of multi-omics approaches (proteomics/transcriptomics) to elucidate downstream effectors.

## 5 Conclusion

This report delineates the clinical and molecular landscape of *SIAH1*-associated NDDs, emphasizing the critical role of zinc finger domain integrity in neurodevelopment. The p.Phe96Leu variant expands the mutational spectrum and underscores the need for comprehensive functional characterization of domain-specific variants. Our findings advocate for *SIAH1* inclusion in diagnostic gene panels for patients presenting with developmental delay, dysmorphic features, and multisystem anomalies.

## Data Availability

The datasets presented in this study can be found in online repositories. The names of the repository/repositories and accession number(s) can be found in the article/Supplementary material.
